# Reduced cervical cancer screening in Zimbabwe as an indirect impact of the COVID-19 pandemic: implications for prevention

**DOI:** 10.11604/pamj.2021.38.131.27852

**Published:** 2021-02-05

**Authors:** Grant Murewanhema

**Affiliations:** 1 Department of Obstetrics and Gynaecology, University of Zimbabwe, College of Health Sciences, P.O Box A172, Avondale Harare, Zimbabwe

**Keywords:** COVID-19, cervical cancer, screening, Zimbabwe

## Abstract

The COVID-19 pandemic has brought unprecedented challenges to healthcare for women, including disruption of cervical cancer screening services. Zimbabwe is a high cervical cancer burden country, with the cancer being the leading malignancy among women. The disruptions in screening could have long-term negative impacts on cervical cancer burden reduction in Zimbabwe. Factors related to institutions, policy, clients and healthcare workers have contributed to the decline in screening and need to be addressed urgently to restore this essential service.

## Perspectives

The first cases of COVID-19 in Zimbabwe were reported in March 2020 [1]. The government introduced multi-pronged approaches to curb the spread of SARS-CoV-2, prepare the health system for possible exponential outbreak growth, and reduce the adverse socio-economic consequences of the pandemic [2]. Reports suggest that women and girls are vulnerable, and may suffer disproportionately from the indirect effects of the pandemic [3-5]. As financial and human resources are channelled more towards COVID-19 responses, both by governments and development partners, critical aspects of women´s health may be neglected [2,6]. Unfortunately, the long-term impacts of this neglect may be more devastating than the pandemic itself [3]. Zimbabwe has a high burden of cervical cancer [7,8]. According to the Zimbabwe National Cancer Registry, this cancer leads among malignancies in women, and constituted 33.2% of the cancer burden in this population with 1308 cases in 2016 [7]. However, despite the fact that Zimbabwe has a well-established national cancer registry, these statistics may still be underestimates. Anecdotal evidence suggests that many women die of cervical cancer without histological confirmation or seeking appropriate treatment, thus they may never make their way into the national cancer registry. It is now established that persistent high-risk Human Papilloma Virus infections, which are vaccine preventable, cause over 99% of cervical cancer cases [9]. However, secondary prevention, which entails regular screening, has been the crux of cervical cancer prevention in Zimbabwe [10]. Considerable progress has been realised towards establishing working cervical cancer screening programmes in Zimbabwe. However, the COVID-19 pandemic may indirectly reverse the gains, leading to missed opportunities for secondary prevention and detection of early stage disease. As health facilities including hospitals and clinics shifted to emergency mode to protect the healthcare workers (HCWs) and clients from contracting COVID-19, cervical cancer screening activities were stopped, alongside other essential components of sexual and reproductive health (SRH). I briefly describe the set-up of the cervical cancer screening programme in Zimbabwe, discuss the possible impact of the COVID-19 pandemic on cervical cancer screening, and offer recommendations for restoration and continuity of this critical service.

**The set-up of the cervical cancer screening programme in Zimbabwe:** health centres located throughout the country offer visual inspection of the cervix with acetic acid cervicography (VIAC) supported by the Ministry of Health and Child Care (MOHCC) and development partners. Nurses are trained to provide this service with the aim of differentiating grossly normal from abnormal cervices. They offer cryotherapy or refer to higher facilities for further evaluation in uncertain or suspicious cases. Conventional Pap smears are offered in some clinics supported by development partners and at hospitals. Testing for high-risk HPV genotypes is offered through development partners at subsidised costs. Colposcopy services are available at tertiary hospitals, where referred patients are biopsied and offered appropriate treatment. The linkage to care, though not formally evaluated, seems to be working. Parallel screening structures exist in the private sector, but the majority of at-risk women may have no medical insurance and do not afford private care.

**Effects of the COVID-19 pandemic on cervical cancer screening:** whilst there has been no formal evaluation of the impact of the pandemic on cervical cancer screening to date, postulates can be drawn. The initial response to the pandemic was total shutdown of all services with the exception of critical healthcare and security services [1,2]. No cervical cancer screening was happening at this time. The lockdowns have been gradually relaxed, but screening services in most centres have remained minimal. Healthcare workers cite lack of adequate personal protective equipment (PPE) to safely and confidently discharge duties, especially for services considered non-critical [11]. Evidence emanating from other settings suggests heightened fear and anxiety as contributory factors to non-restoration of services [12]. Some centres may also be experiencing shortages of consumables for screening. The COVID-19 pandemic is widely reported to have disrupted supply chains, which may result in increasing costs for importing consumables [13]. Client factors contribute to reduced utilisation of services. Individuals who are well may not visit health facilities for fear of contracting COVID-19. An unpublished survey conducted by the risk communication and community engagement pillar noted fear of contracting the disease, fear of being stigmatised as COVID-19 cases and other fears emanating from lack of knowledge as barriers to utilisation. Movement restrictions, transport challenges and poor communication emanating from bad roads and digital networks may further compound reduced health facility visits [11].

**Implications for cervical cancer prevention:** the duration of the pandemic is unpredictable; therefore, clients may not be electively seeking cervical cancer screening services for a long time to come. In a country with a high human immunodeficiency virus (HIV) burden, a risk factor for the high prevalence of cervical cancer, many missed opportunities for prevention exist due to the COVID-19 pandemic. Patients with potentially curable early-stage disease may eventually present with more advanced disease, resulting in higher morbidity and mortality. Advanced cancer requires more expensive and toxic multimodality treatments, and Zimbabwe has struggled with radiotherapy machines, which are only found at the oncology unit in Harare. The cost of cytotoxic medicines is also quite substantial for a fragile economy. Long-term cost-effectiveness analyses of reduced cervical cancer screening must be seriously considered. Screening is not occurring at a time when HPV vaccination services for adolescent girls are interrupted. Human immunodeficiency virus care and treatment services, which are a gateway to cervical cancer screening for the HIV-infected are also disrupted, and some people living with HIV and AIDS (PLWHA) may be encountering disruptions to their treatment [14-16]. Zimbabwe has an estimated 1.3 million PLWHA [14]. Modelling estimates have predicted excess HIV-related deaths in Sub-Saharan Africa in the next four years if disruptions to service provision occur and continue [15]. A healthcare system that was reported as fragile before the onset of the pandemic may emerge weaker at the end, and fail to cope with increased disease burden [2]. The prevailing health system and its organisation in Zimbabwe presents significant barriers to the uptake of cervical cancer care and treatment [8], necessitating the need to restore preventive health services urgently, including cervical cancer screening. Teaching services for undergraduate and postgraduate medical students were negatively impacted. Unfortunately, cervical cancer screening is a transferrable skill, and a protracted duration of reduced training will result in inadequate on-job training for future practitioners. Other HCWs involved in screening also need continuous exposure to the procedure; otherwise efficiency may be lost over time.

**Recommendations for restoring cervical cancer screening services during the pandemic:** alongside other SRH services, cervical cancer screening must be restored. However, the safety of both HCWs and clients must come first. It is of paramount importance to put in place adequate infection prevention and control (IPC) measures before restoring services. The WHO guidance on maintaining essential services during the COVID-19 pandemic provides useful guidance on rationalising the use of PPE in resource-limited settings [17], and contextualised adaptation may be considered. Frameworks for effectively addressing the identified domains must be put in place to encourage restoration of essential cervical cancer screening services and reduce the burden of cervical cancer. Recommendations for restoring cervical cancer screening services during the COVID-19 pandemic are given in Table 1.

**Table 1:** recommendations for restoring cervical cancer services during the pandemic in Zimbabwe

LEVEL	Domain	Action/recommendation
**Institutional**	**Space**	Identify and reserve a spacious area for cervical cancer screening which promotes physical distancing.
		This, like already exists in some unit, can be integrated within sexual and reproductive health units.
	**Infection, prevention and control**	Address issues of adequate PPE for cervical cancer screening.
		Recommended by the WHO is a medical mask and an apron, considering that cervical cancer screening is a non-aerosol generating procedure.
		Provide hand washing ports at all screening facilities, and basic temperature checks
**Client/patient**	**Fear**	Correct messages through accessible media, including social media to dispel rumours, myths and misconceptions regarding COVID-19.
	**Accessibility**	Bring cervical cancer screening activities to clinics nearest to where people stay, especially in the more marginalised areas.
		Development partners, Ministry and local leadership including community leaders can identify space for setting up screening facilities, alongside other components such as family planning.
**Policy**	**Risk communication and community engagement**	Critical to educate communities regarding their risk of acquiring COVID-19 at facilities versus in the community. The risk of contracting COVID-19 in the community may be higher than in facilities with adequate IPC measures.
		Information, Education and Communication material must integrate COVID-19 messages with other essential SRH messages, including cervical cancer screening.
	**HCW welfare**	Urgently restore remuneration and risk allowances for healthcare workers.
		Life insurance packages that are attractive for healthcare workers help to alleviate anxiety and boost morale.
**Academic**	**Training**	Conditions that promote safe teaching and continuous transfer of skills must be put in place urgently.
		Adopt video-conferencing based teaching methods.
	**Monitoring and evaluation/operational research**	Operational research to document the impact of the pandemic on cervical cancer epidemiology including screening.

## Conclusion

Restoration of cervical cancer screening in Zimbabwe is critical, to prevent an emerging increase in invasive cervical cancer burden. A multi-stakeholder approach is needed to restore services in an environment that ensures the safety of clients and healthcare workers. Therefore, the government, development partners, the communities and local authorities must work together to develop implementable and affordable interventions to ensure a continuum of cervical cancer preventive services during the ongoing COVID-19 pandemic.
